# Case report: Resolution of VIPoma-related symptoms with peptide receptor radionuclide therapy

**DOI:** 10.3389/fonc.2024.1432758

**Published:** 2025-01-08

**Authors:** Turgut Bora Cengiz, Raksha Kulkarni, Virginia Corbett, Nasrin V. Ghesani, Edward Wolin, Munir V. Ghesani

**Affiliations:** ^1^ Department of Nuclear Medicine, Mount Sinai Hospital at Icahn School of Medicine, New York, NY, United States; ^2^ Department of Hematology and Oncology, Mount Sinai Hospital at Icahn School of Medicine, New York, NY, United States

**Keywords:** PRRT, VIPoma, inpatient PRRT, neuroendocrine tumor, pancreatic net, 68Ga DOTATATE PET/CT, theranostics

## Abstract

Peptide receptor radionuclide therapy (PRRT) is used for the management of neuroendocrine tumors (NETs) not responsive to somatostatin analogs. In this case series, we report two patients with pancreatic vasoactive intestinal peptide (VIP)-secreting NETs (VIPomas) not responsive to any other therapies who achieved symptomatic control and a significant decrease in serum VIP levels with PRRT during their hospital stay. Two patients with VIPomas were admitted to the hospital with multiple prior hospital admissions after going through multiple lines of therapy. The first patient was a 47-year-old woman with a grade 2 pancreatic VIP-secreting NET. She was treated with somatostatin analogs and chemotherapy; however, she experienced recurrent symptoms and complications leading to two hospital admissions, one of which included an intensive care unit (ICU) admission. The patient was treated with lutetium-177 DOTATATE while in the hospital, which led to the resolution of the symptoms and a marked decline in serum VIP levels, and she was able to be discharged from the hospital after 147 days of hospitalization (16 days after PRRT). The second patient was a 27-year-old man who was diagnosed with a well-differentiated grade 3 pancreatic VIPoma. He also suffered from severe diarrhea and was not responsive to any form of therapy, including liver embolization. He was also treated with PRRT on admission, which led to immediate resolution of his symptoms and a decrease in tumor markers. In conclusion, ^177^Lu-DOTATATE is an effective treatment for highly symptomatic VIPoma. Inpatient administration of PRRT can rapidly reduce diarrhea and fluid and electrolyte complications of VIP secretion and may shorten hospital stays.

## Introduction

Functional pancreatic tumors, although less common than small bowel neuroendocrine tumors (NETs), can have a profound impact on patients’ quality of life. There is a paucity of data on functional pancreatic NETs and their treatment response to peptide receptor radionuclide therapy (PRRT).

PRRT has been proven to improve progression-free survival in gastroenteropancreatic NETs (1–3). The utility of PRRT in halting disease progression and achieving symptomatic relief has been studied extensively. However, the majority of studies have included patients with small bowel NET. Pancreatic NET can also produce a variety of symptoms that can lead to life-threatening electrolyte and hormonal imbalances, and there are limited data in the literature regarding the role of PRRT in functional pancreatic tumors. In this study, we report the outcomes of two patients who were diagnosed with vasoactive intestinal peptide-producing tumors (VIPoma), who were extremely symptomatic, had electrolyte imbalances, and experienced dramatic resolution of their VIPoma syndromes with PRRT.

## Case presentations

### First case

A 47-year-old female patient presented to the hospital with complaints of sudden epigastric and flank pain. She was found to have a 1.3-cm right renal calculus and innumerable liver masses along with a 12.6-cm nodular conglomerate in the periportal region compressing the splenic, portal, and left renal veins and the common bile duct on a CT scan. She underwent right ureteral stent placement and a percutaneous liver biopsy. The biopsy revealed a NET G2 with Ki-67 of 5%. The biopsy specimen was negative for insulin, somatostatin, and glucagon.

**Figure 1:**
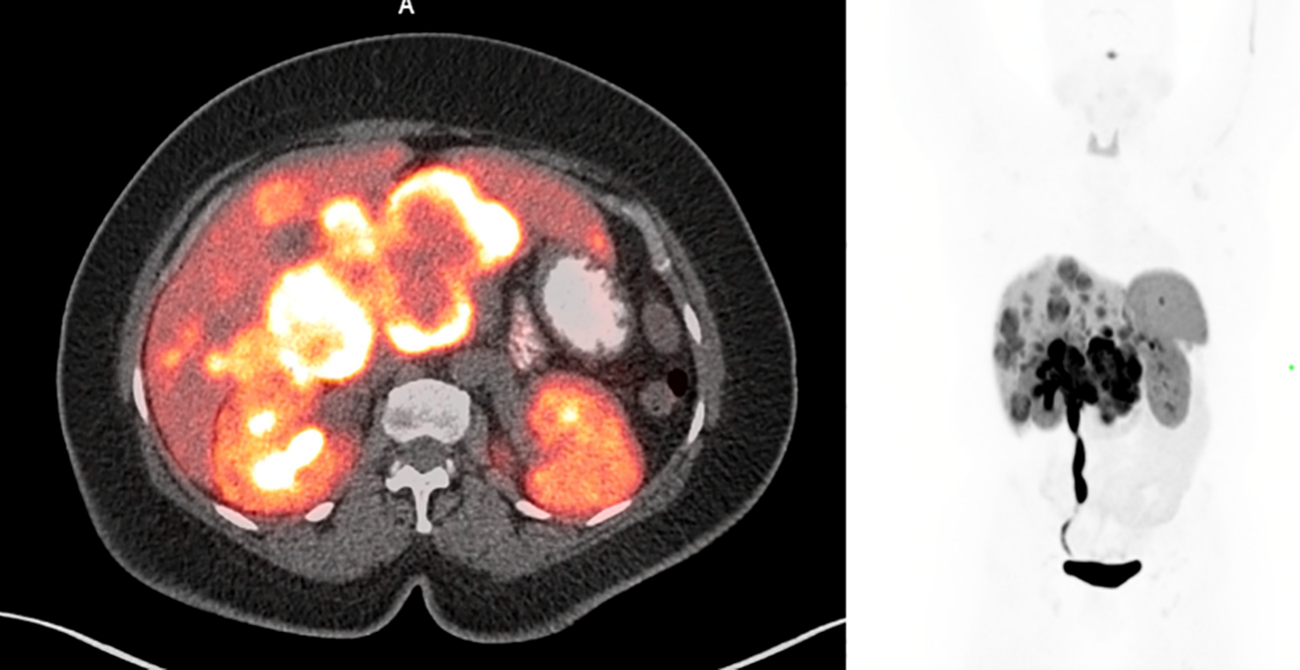
Pre-treatment ^68^Ga-DOTATATE PET/CT showing multiple tracer avid liver lesions and a peripancreatic mass. Peripancreatic mass SUVmax was 36.7.

Magnetic resonance imaging (MRI) of the abdomen showed a peripancreatic mass, multiple bilobar liver metastases, and right-sided hydronephrosis. The patient then underwent PET/CT with ^68^Ga-DOTATATE, which demonstrated intense tracer avidity of the periportal mass and multiple tracer avid liver lesions (Krenning score 4). The DOTATATE PET/CT can be seen in **Figure 1**.

The patient initially complained of nausea, hot flashes, and bloating after meals.

#### Initial treatment course

After the initial staging, the patient was started on lanreotide 120 mg and a combination of capecitabine and temozolomide. Three months later, an MRI of the abdomen and pelvis showed enlargement of one of the liver lesions compared to the MRI performed 4 months earlier. Her right-sided hydronephrosis had resolved by this time. During this visit, the patient described an unintentional weight loss of 20 lbs. along with three to five watery bowel movements per day. The patient’s VIP level was 676.9 pg/mL (reference range 0–58.8 pg/mL). The patient’s symptoms of multiple episodes of watery diarrhea, elevated VIP levels, and negative staining for insulin, glucagon, and somatostatin confirmed the diagnosis of VIPoma.

**Figure 2:**
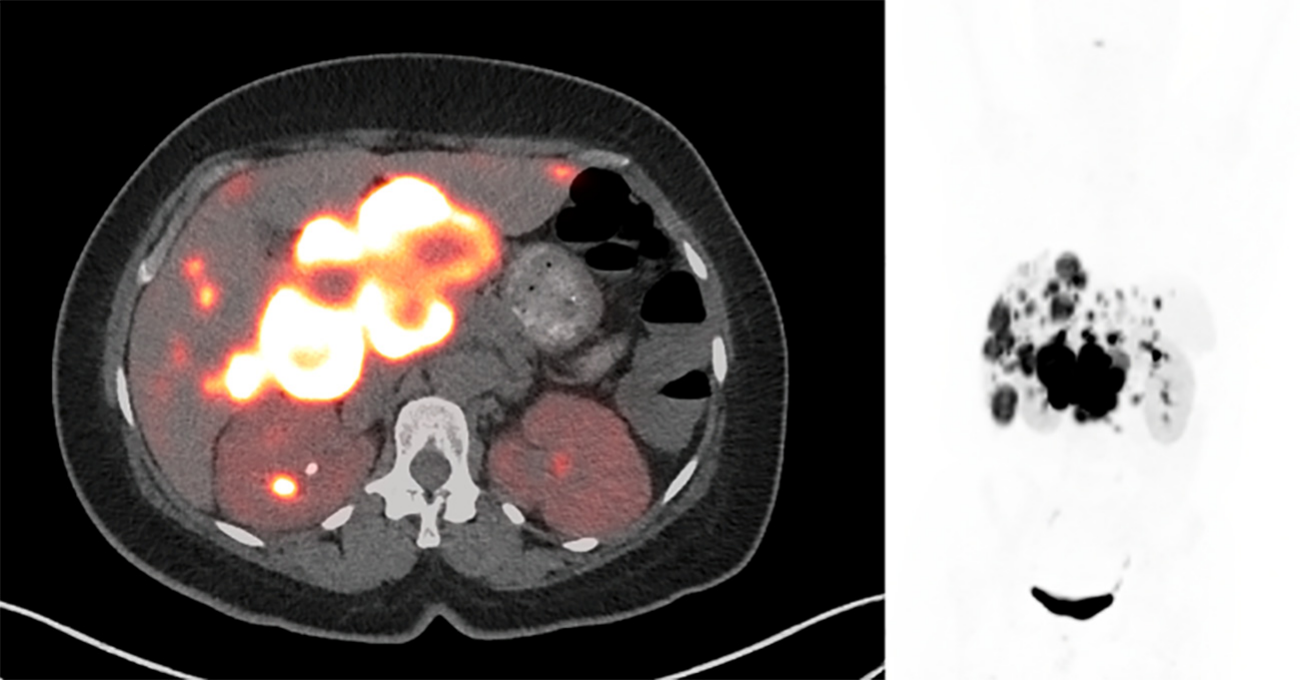
Post-chemotherapy ^68^Ga-DOTATATE PET/CT shows intense radiotracer uptake in the peripancreatic mass and liver lesions, which was not significantly changed compared to pre-treatment PET/CT. SUVmax of the peripancreatic mass was 82.4. There was interval resolution of the prominent activity in the right urinary collecting system.

Despite the chemotherapy, the patient’s watery diarrhea rapidly progressed. There were no significant changes on the post-chemotherapy images (**Figure 2**). The chemotherapy was then stopped, and the patient was referred to nuclear medicine for PRRT. PRRT with ^177^Lu-DOTATATE was planned; however, the patient’s intractable nausea, vomiting, and diarrhea worsened, which led to an emergency department (ED) visit.

#### First hospitalization

The patient was hospitalized for 6 days with bilious vomiting, watery diarrhea, and inability to tolerate oral nutrition. At that time, she had hypokalemia 2.7 MEq/L (reference range 3.5–5.2), elevated creatinine 1.71 (reference range 0.5–1.1), and metabolic acidosis. The patient was discharged after 3 months of hospitalization on total parenteral nutrition (TPN) and subcutaneous octreotide in anticipation of receiving outpatient PRRT.

#### Second hospitalization

**Figure 3:**
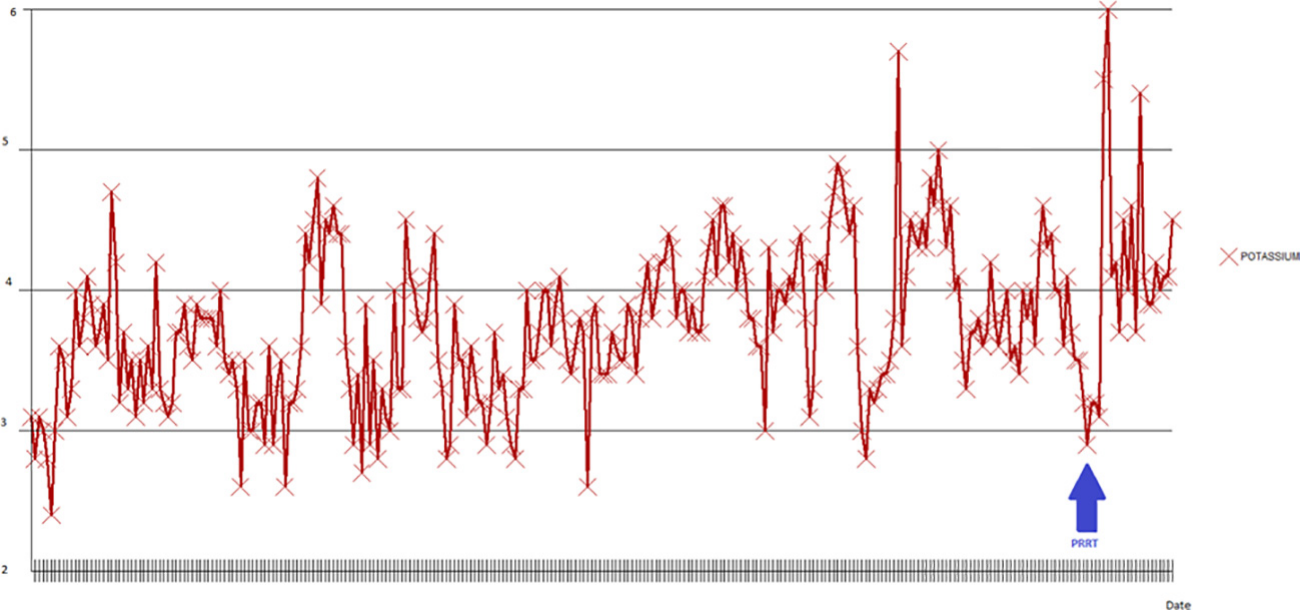
Potassium changes during the second hospitalization. The lowest potassium level measured was 2.4 MEq/L in the early phases of admission (blue arrow).

The patient was readmitted to the hospital 2 weeks after her discharge with gastrointestinal bleeding and worsening watery diarrhea associated with an increase in the VIP level to 33,309 pg/mL. Upper endoscopy did not show any source of bleeding, but there was bulging of her NET into the duodenal bulb. While in the hospital, IV fluids, TPN, and correction of electrolyte imbalances were required along with an octreotide drip of 200 mcg/hr. Trials with other agents such as interferon alpha and everolimus showed no benefit. She developed bacteremia and fungemia, which were treated appropriately. The patient was admitted to the intensive care unit (ICU) with acute worsening of her general status and severe hypokalemia on the 51^st^ day of her hospital stay. The patient’s changes in serum potassium can be seen in **Figure 3**. Due to the patient’s intractable diarrhea and severe electrolyte imbalance, the treatment team elected to administer PRRT during the hospital stay.

#### PRRT

**Figure 4:**
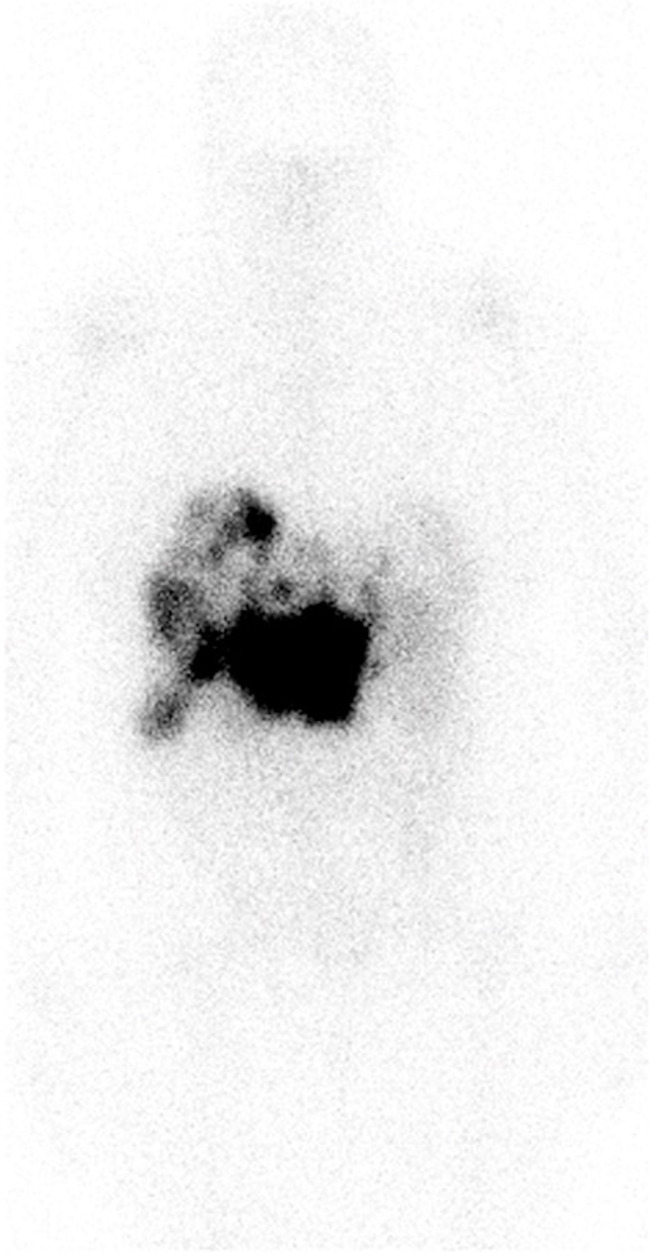
Anterior images of post-treatment lutetium-177 DOTATATE images obtained 5 days after PRRT. Intense uptake was seen in the bulky mass in the peripancreatic region with multiple liver metastases. Of note, octreotide infusion was performed 8 h before PRRT. PRRT, peptide receptor radionuclide therapy.

**Figure 5:**
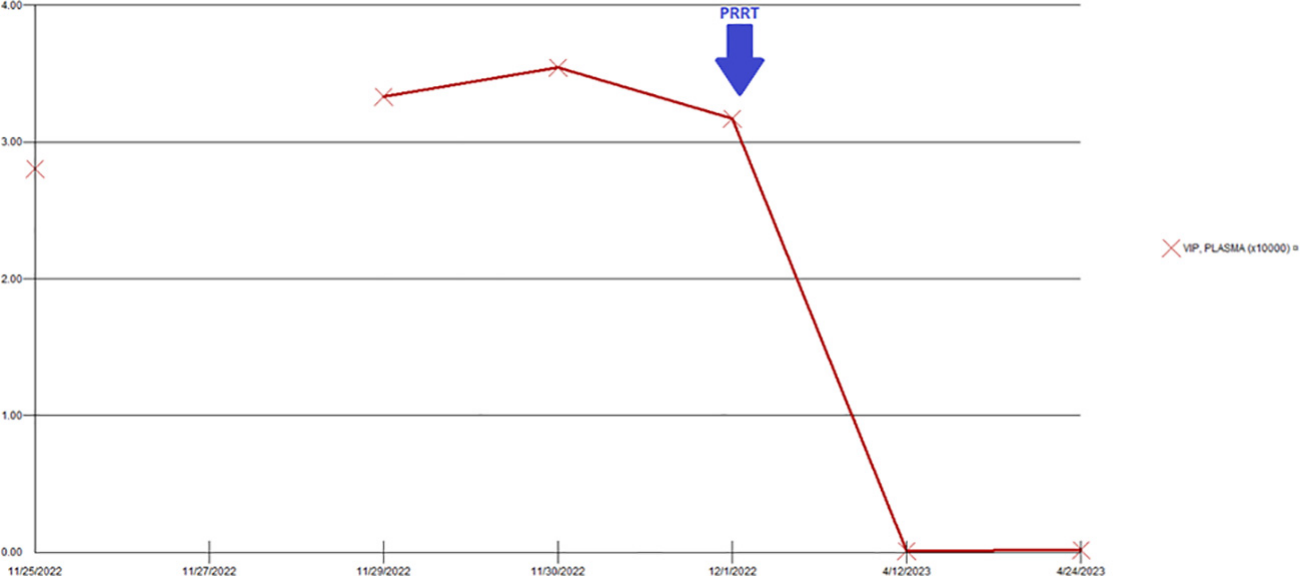
Changes in serum VIP levels during the hospitalization. There was a significant decrease in the VIP level after administration of PRRT. VIP, vasoactive intestinal peptide; PRRT, peptide receptor radionuclide therapy.

The patient was moved to a room with lead shielding in preparation for PRRT. Nuclear medicine physicians and radiation safety officers briefed the nurses and the ancillary staff caring for the patient about radiation safety protocols. The octreotide infusion was held for 8 h, and on the 131st day after admission, the patient was treated with 200 mCi (7.4 GBq) of ^177^Lu-DOTATATE with 1 L of l-lysine and l-arginine solution. The patient remained on electrolyte supplements after PRRT due to ongoing hypokalemia, and daily laboratory tests were handled per the radiation safety protocols. On the third day after PRRT, the patient reported a significant decrease in her watery diarrhea and nausea. A post-therapy image was obtained on the fifth day after PRRT and can be seen in **Figure 4**. On post-treatment day 8, the patient was switched to subcutaneous octreotide (500 mcg QID), and her oral intake returned to normal. There was a marked decrease in serum VIP levels at this time from a peak of 35,457 pg/mL to 108.9 pg/mL on post-treatment day 14. The serum VIP graph can be seen in **Figure 5**.

The patient was deemed fit for discharge 147 days after admission (16 days after PRRT). At the time of discharge, she was having one to two soft bowel movements per day; her nausea and abdominal pain had resolved; and she was on 120 mg of subcutaneous lanreotide. The patient was scheduled to receive her second dose of PRRT approximately 8 weeks after her initial therapy. The patient did not demonstrate any changes in hematological or metabolic parameters.

### Second case

A 27-year-old man presented with decreased appetite and fatigue. On initial evaluation, CT of the abdomen with contrast showed a 2.2-cm pancreatic lesion and multiple hepatic lesions suggestive of metastatic disease from the primary pancreatic tumor. An endoscopic ultrasound-guided fine-needle aspiration biopsy of the pancreas revealed a well-differentiated NET G3 (Ki-67 40%). Immunohistochemistry was positive for synaptophysin, chromogranin, and cytokeratin AE1/AE3, with a low mitotic rate. He also had multiple episodes of diarrhea requiring electrolyte support. VIPoma was one of the differentials in addition to carcinoid syndrome, given the high 5-hydroxy indole acetic acid (5-HIAA) and serotonin levels. He was started on carboplatin and etoposide. Serum VIP level after the initial chemotherapy was 523 pg/mL and slowly rose to 1,369.8 pg/mL. His chemotherapy course was complicated by intractable diarrhea. The patient was then diagnosed with VIPoma.

**Figure 6:**
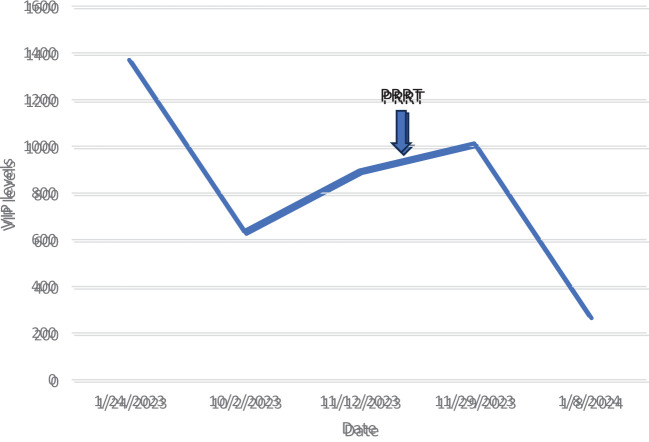
VIP levels over time in case 1. VIP, vasoactive intestinal peptide.

A ^68^Ga-DOTATATE PET/CT after chemotherapy showed multiple liver metastases, a pancreatic lesion, and a retroperitoneal nodule (**Figure 6**).

#### Subsequent course of treatment

The patient then received octreotide for 1 month without any symptom relief, followed by 5-fluorouracil and irinotecan. Urine 5-HIAA was 112.8 mg/24 h, chromogranin was 6,696 ng/mL, and the VIP level was 573 pg/mL at this time. The patient then received lanreotide, followed by capecitabine and temozolomide for 3 months.

The patient then underwent multiple bland embolizations, with some symptom reduction.

He then received sunitinib, which was discontinued after one cycle due to renal dysfunction. At this time, the patient was hospitalized for over 1 month due to dehydration and severe electrolyte imbalance. A follow-up MRI showed a reduction in the size of the liver metastases; however, new osseous metastases were detected, indicating the progression of the disease.

**Figure 7:**
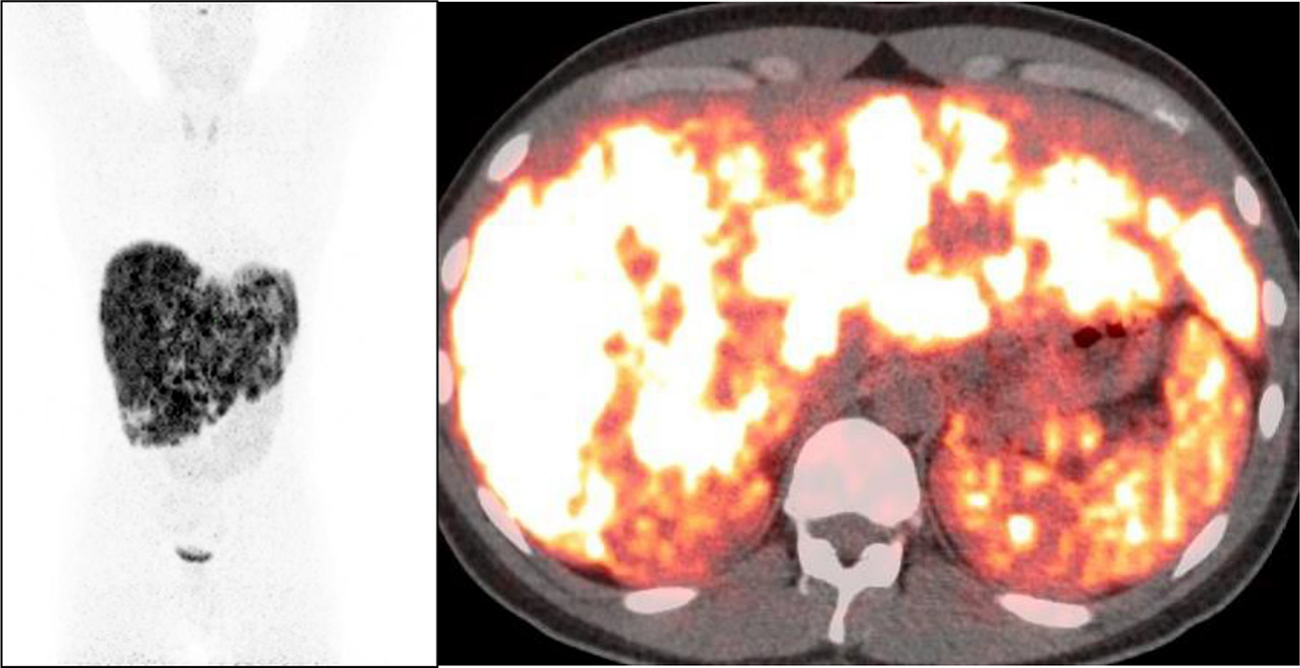
VIP levels over time and after PRRT for case 2. VIP, vasoactive intestinal peptide; PRRT, peptide receptor radionuclide therapy.

A follow-up ^68^Ga-DOTATATE PET/CT showed post-embolization changes in the liver and no new liver metastases. There was a decrease in the size of the pancreatic tail mass and resolution of the left retroperitoneal lymph node (**Figure 7**).

The patient was once again admitted to the hospital with intractable diarrhea, hypotension, electrolyte imbalance, and decreased oral intake. His plasma 5-HIAA was 130 ng/mL, and chromogranin A was 8,119 ng/mL at the time of admission.

#### PRRT

Considering the progression and uncontrolled symptoms after multiple lines of therapy, the consensus was to proceed with PRRT during hospitalization. The patient was on an octreotide drip, which was discontinued 6 h before the PRRT. He then received the first dose of PRRT at 197.4 mCi. The procedure of administering the amino acids, radioligand therapy, and radioactive safety precautions was similar to those used for the first patient. After the infusion of 177LuDOTATATE was completed in the Nuclear medicine department, the patient was moved back to their lead shielded room to complete the amino acid infusion.

The patient was restarted on an octreotide drip after his treatment. His hypokalemia, hypomagnesemia, and hypophosphatemia resolved soon after. The patient’s bowel movements were reduced to two to three per day. He was discharged from the hospital 2 weeks after the PRRT.

**Figure 8:**
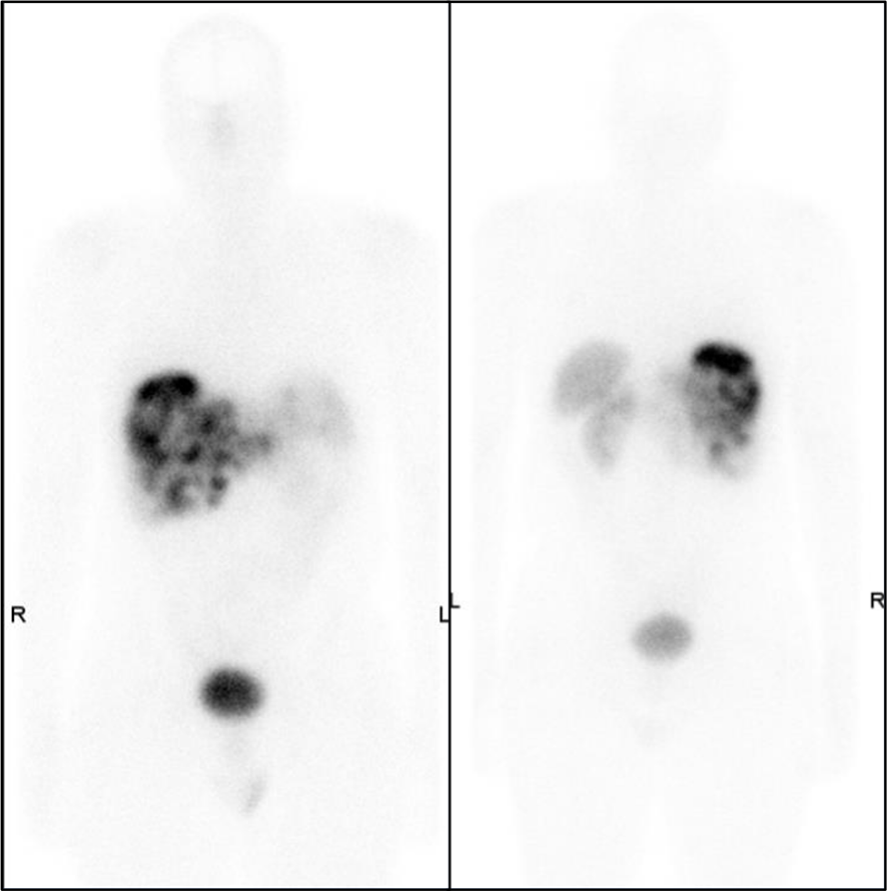
Post-chemotherapy ^68^Ga-DOTATATE PET/CT showing multiple tracer avid liver lesions and a peripancreatic mass.

His chromogranin level decreased to 824 ng/mL (from 8,119 ng/mL) 1 month after PRRT, and the serum VIP level fell to 247 pg/mL (**Figure 8**) from its peak of 1,369.8 pg/mL.

At follow-up following the first cycle of PRRT, the patient reported two to three loose stools per day. The patient reported that his energy was markedly improved, and his appetite was better. His pain had resolved, reducing the need for fentanyl patches. His maintenance therapy was continued with subcutaneous octreotide. The patient has completed three cycles of PRRT to date without any significant adverse effects on hematological or metabolic parameters.

His general condition has continued to improve; he has two to three semi-solid bowel movements per day; his pain has resolved; and he has resumed work.

## Discussion

In this study, we demonstrated that PRRT can rapidly reduce the hormonal secretion and associated symptoms of excess VIP production from VIPoma. In our case series, two patients suffered life-threatening electrolyte imbalances due to persistent vomiting and watery diarrhea, which caused prolonged hospital stays and an ICU admission before achieving symptomatic control with PRRT. It is possible that earlier implementation of PRRT could prevent the detrimental effects of VIPomas and other functional pancreatic tumors.

NET is a spectrum of disorders with unique presentations based on the tumor subtype when they are functional, yet the majority of NETs are non-functional tumors. While there are compelling data on carcinoid syndrome and PRRT in the literature, functional pancreatic NET is a rare condition that has multiple subtypes such as gastrinomas, insulinomas, and glucagonomas. One study analyzed over 1,400 cases of pancreatic NET and found that 90.8% of the NETs were non-functional (4). Almost 1% of the whole cohort had malignant VIPoma, which makes VIP-secreting tumors a rare entity even within the subgroup of functional pancreatic NETs.

Being a unique molecule, VIP is mainly produced in the duodenum and delta-2-pancreatic islet cells and has a short half-life (2 minutes) for a peptide hormone (5, 6). VIP is commonly found in the central and peripheral nervous systems with regulatory effects on gastric acid secretion and cell motility, but most importantly on intestinal peristalsis and vasodilation (6, 7). It is thought that the watery diarrhea caused by VIPomas is secondary to its prosecretory effect leading to anion secretion (mainly Cl^−^) through its action on VPAC1 receptors in the intestinal mucosa, which drives water into the small bowel lumen following Cl^−^. Hypersecretion of VIP can also lead to life-threatening hypokalemia and non-anion gap acidosis (8). Watery diarrhea (usually up to 10 bowel movements per day) is seen in almost 90% of the patients as a consequence of VIP-activated intestinal epithelial cells leading to upregulation of cAMP even during fasting periods (9, 10). The nature of excess hormone release and the related intractable diarrhea can be addressed with long-acting somatostatin analogs with loperamide. In our case, the first patient suffered long-standing watery diarrhea leading to severe hypokalemia as low as 2.4 MEq/L, which was unresponsive to octreotide drip at maximal doses with anti-diarrheal support. Severe dehydration and metabolic disturbances can predispose patients to nosocomial infections, which may ultimately delay tumoricidal therapy. It is also very important to have diarrhea under control for radiation safety purposes before PRRT, despite only a small fraction of ^177^Lu-DOTATATE being cleared via the gastrointestinal system.

The role of PRRT in progression-free survival in NETs has been established (11, 12). The role of PRRT in pancreatic NET is also growing, with studies showing improved outcomes (12, 13). The majority of the studies aimed to assess the association of PRRT and progression-free or overall survival in either small bowel or pancreatic non-functional NET. Symptomatic response in hormone-producing functional pancreatic NET is yet to be fully assessed given its significant impact on patients’ quality of life. Only a few studies have shown the efficacy of PRRT in hormone-secreting pancreatic NET (14). A recent study evaluating insulinoma patients treated with either ^90^Y or ^177^Lu-DOTATOC showed that 58% of the patients were able to reduce their anti-hypoglycemic medications (15). One study analyzed 34 patients with metastatic functional pancreatic NET (5 with VIPoma) and found that PRRT could achieve symptomatic relief in 71% of the patients who had uncontrolled symptoms at the baseline (16). Of these five patients with VIPoma, 80% had experienced symptomatic relief after PRRT. Another study evaluated 15 patients with VIPomas. Six of 15 (40%) of the patients received ^177^Lu-DOTATATE. Five of six (83.3%) achieved significant resolution of symptoms on monotherapy with ^177^Lu-DOTATATE, while one patient needed the concomitant use of somatostatin analogs (17). While the majority of the literature on the use of ^177^Lu-DOTATATE in patients with VIPoma indicates an overall favorable response to this therapy, a few reports mention poor tolerance to ^177^Lu-DOTATATE (18, 19). Although the literature is limited in terms of VIPoma and the efficacy of PRRT in eliminating the hormone-related symptoms, our case series in conjunction with the above study shows that symptomatic relief in VIPoma can be achieved with PRRT (**Table 1**).

**Table 1:** Studies demonstrating response to PRRT in patients with VIPomas.

Authors, year	Paper	Number of patients	Patients with VIPoma	Symptom control	Outcomes				PFS	Adverse events
					PR	CR	PD	SD		
Zandee WT et al. (16)	Retrospective	34	5	4	4	0	1	0	N/A	Patients with PD developed severe diarrhea.
Angelousi et al. (17)	Retrospective	6	6		6	0	0	0	26 months	
Kwekkeboom et al. (20)	Retrospective	91	2	N/A	1	0	0	1	N/A	N/A
Audil, Y Hadiyah (21).	Case report	1	1	1	1	–	–	–	N/A	None

PR, partial response; CR, complete response; PD, Progression of disease; SD, stable disease.

In addition to the above studies describing the use of PRRT in VIPomas, several studies have discussed the use of ^177^Lu-DOTATATE in functional pancreatic tumors as a group and its utility in symptom control, palliation, and improvement of quality of life (22, 23) (**Table 1**). To the best of our knowledge, there are no reports of inpatient administration of ^177^Lu-DOTATATE in the United States.

Our study showed that excess VIP secretion can rapidly become life-threatening. PRRT is a valuable option in metastatic VIPoma; however, the cost of PRRT is the main drawback for in-hospital administration in the United States. In our case series, we demonstrated that the benefits of PRRT may outweigh the financial concerns and could have prevented the complications of VIPoma secretion and the associated downstream costs of hospitalization. In such cases, providers may consider single-case agreements (single-patient contracts) to overcome the high financial burden of PRRT, which is lower than the cost of multiple hospital admissions, or prolonged hospital stays requiring intensive care.

This report shows that PRRT provides excellent symptomatic control in patients with metastatic VIPoma and a drastic decrease in serum VIP. Providers should consider PRRT in functional pancreatic NET in hospitalized patients despite financial concerns associated with the index cost of PRRT.

## Data Availability

The original contributions presented in the study are included in the article/supplementary material. Further inquiries can be directed to the corresponding author.
